# Exposure to a sensory functional ingredient in the pig model modulates the blood-oxygen-level dependent brain responses to food odor and acute stress during pharmacological MRI in the frontostriatal and limbic circuits

**DOI:** 10.3389/fnut.2023.1123162

**Published:** 2023-02-28

**Authors:** Emmanuelle Briard, Yann Serrand, Patrice Dahirel, Régis Janvier, Virginie Noirot, Pierre Etienne, Nicolas Coquery, Pierre-Antoine Eliat, David Val-Laillet

**Affiliations:** ^1^INRAE, INSERM, Univ Rennes, Nutrition Metabolisms and Cancer, NuMeCan, St Gilles, Rennes, France; ^2^Phodé, Terssac, France; ^3^CNRS, INSERM, Biosit UAR 3480 US_S 018, PRISM, Univ Rennes, Rennes, France

**Keywords:** functional food ingredients, brain, behavior, fMRI, olfaction, pharmacological MRI

## Abstract

**Introduction:**

In the present study, we examined the effects of a supplementation with a sensory functional ingredient (FI, D16729, Phodé, France) containing vanillin, furaneol, diacetyl and a mixture of aromatic fatty acids on the behavioural and brain responses of juvenile pigs to acute stress.

**Methods:**

Twenty-four pigs were fed from weaning with a standard granulated feed supplemented with the functional ingredient D16729 (FS animals, *N* = 12) or a control formulation (CT animals, *N* = 12). After a feed transition (10 days after weaning), the effects of FI were investigated on eating behaviour during two-choice feed preference tests. Emotional reactivity to acute stress was then investigated during openfield (OF), novel suddenly moving object (NSO), and contention tests. Brain responses to the FI and the two different feeds’ odour, as well as to an acute pharmacological stressor (injection of Synacthen®) were finally investigated with functional magnetic resonance imaging (fMRI).

**Results:**

FS animals tended to spend more time above the functional feed (*p* = 0.06) and spent significantly more time at the periphery of the arena during NSO (*p* < 0.05). Their latency to contact the novel object was longer and they spent less time exploring the object compared to CT animals (*p* < 0.05 for both). Frontostriatal and limbic responses to the FI were influenced by previous exposure to FI, with higher activation in FS animals exposed to the FI feed odor compared to CT animals exposed to a similarly familiar feed odor without FI. The pharmacological acute stress provoked significant brain activations in the prefrontal and thalamic areas, which were alleviated in FS animals that also showed more activity in the nucleus accumbens. Finally, the acute exposure to FI in naive animals modulated their brain responses to acute pharmacological stress.

**Discussion:**

Overall, these results showed how previous habituation to the FI can modulate the brain areas involved in food pleasure and motivation while alleviating the brain responses to acute stress.

## Introduction

Functional food ingredients derived from natural extracts are of growing interest in human and animal nutrition to optimize food intake and improve well-being through adaptation to potential stressors for example (1). Usually composed of aromatic substances (essential oils, aromatic herbs, spices, etc.), these functional ingredients have been found to be interesting alternatives to antibiotics, have antimicrobial and antioxidant properties (2, 3) as well as an effect on growth (4, 5), hankering (6) and behavior (7–9).

In previous preclinical studies in a pig model of chronic psychosocial stress characterized by social isolation, environment impoverishment and unpredictability (10), we have shown that functional food ingredients (FI) based on spice extracts (*Curcuma longa L., Piper nigrum L., Capsicum annuum L., and Zingiber officinale L.*) or *Citrus sinensis* could induce food preference and have possible anxiolytic properties, while modulating brain activity and plasticity in regions involved in pleasure, emotion regulation and cognition (1, 11). Indeed, animals that had supplemented feed with a functional ingredient had a lower number of escape attempts within the openfield test arena and a lower latency to eat during the novelty-suppressed feeding test compared to the control group (11). These behavioral tests and neuroimaging investigation, designed to investigate anxiety behavior (12, 13) and the brain responses to stress (11), showed in our chronic stress model some potential anxiolytic effects of these functional ingredients.

Other food ingredients have been shown to have beneficial effects on stress and/or food pleasure. In the context of the present study, we used a functional ingredient containing vanillin, furaneol, diacetyl and a mixture of aromatic fatty acids. Vanillin, one of the most used flavoring agents in the odorant and food industry, has been extensively studied for its anti-depressant/anti-stress effects (14), as well as neuroprotective effects, which might proceed through anti-oxidant and anti-inflammatory activities (15). Vanillin has been shown to reduce depression-like symptoms in a rat model of chronic depression by elevating dopamine and serotonin levels in the brain (16). The effect of vanillin might be due to its antioxidant properties and its action on monoamine neurotransmitters (increasing the level of serotonin and dopamine in brain tissues) (16, 17). The olfactory pathway is necessary because no similar effects were observed in the olfactory bulbectomy-induced animal model (16). Inhalation of 4-hydroxy-2,5-dimethyl-3(2H)-furanone (DMHF), also known as furaneol®, has been shown to promote appetite in the rat model (18) and was identified as the putative agent responsible for the decrease in alpha brainwave distribution after smelling of a Maillard reaction sample that significantly decreased negative moods (19). DMHF also reduced systolic blood pressure, heart rate and oxidative stress marker levels in rats when inhaled, which highlights again the role of olfaction in modulating not only the mood but also physiological factors related to stress (6). Diacetyl is naturally present in butter and as a bacteria metabolite in fermented products such as beer and white wine. It is a butter-flavoring agent commonly used in the food industry and of which the pleasant odor is easily recognized by consumers. Diacetyl even enters the composition of oral nutritional supplements to increase appetite and pleasure in the elderly for example (20).

In light of this literature, the combination of vanillin, furaneol and diacetyl was consequently thought to be a highly-palatable mixture for food supplementation, capable of increasing appetite and pleasure, regulating mood, and decreasing stress responses, mainly through the olfactory pathway. The aim of the present study was consequently to explore the neurobehavioral effects of a novel functional food ingredient based on a formulation designed and produced by Phodé (France), mainly containing vanillin, furaneol, diacetyl and a mixture of aromatic fatty acids. First, we studied with dedicated behavioral tests a potential reduction of the emotional responses related to acute stress. The two-choice food preference tests, the openfield and restraint tests were chosen according to our experience and previous results obtained in pigs with another food ingredient (11). The novel suddenly moving object (NSO) test was adapted from existing tests in pigs (21, 22) to investigate the emotional reactivity to acute stress and/or fear toward a novel moving object. Second, we used functional magnetic resonance imaging (fMRI) to explore the brain responses to the olfactory perception of feed/ingredient odors as well as to an acute pharmacologically-induced stress.

## Materials and methods

Experiments were conducted in accordance with the current ethical standards of the European Community (Directive 2010/63/EU), Agreement No. C35-275-32 and Authorization No. 35–88 (DVL). The Regional Ethics Committee in animal Experiment of Brittany and the French Ministry of Higher Education and Research have approved and authorized the entire procedure described in this paper (project number APAFIS #31372-2021043015211971_v2).

### Animals, housing and experimental diets

Two batches of 12 Large White/Landrace × Piétrain piglets (six males and six females per group) from the experimental station of the French National Research Institute for Agriculture, Food and Environment (INRAE, Saint-Gilles, France) were used from September to November 2021. Piglets were distributed into two different rooms according to their groups and were housed in individual pens (from weaning to 44 days 80 × 60 × 62 cm, then 120 × 130 × 80 cm).

From weaning, animals of the functional ingredient group (FS, *N* = 12) were fed with a standard granulated feed supplemented with the functional powder ingredient D16729. It contained an active liquid aromatic core mainly based on vanillin, furaneol, diacetyl and a mixture of aromatic fatty acids, and was formulated with glycerine, water, vegetable oil, surfactant, stabilizer and silica. Animals of the standard group (CT, *N* = 12) received a control formulation (D16793: vehicle formulated in powder form), containing glycerine, vegetable oils, water, silica, surfactant, and stabilizer without actives aromatics.

Both products (control and functional) were provided by Phodé (Terssac, France). Animals received a daily feed ration adapted to their weight/age, had unlimited access to water and were subjected to a natural day/night cycle.

### Study design

**Figure 1:**
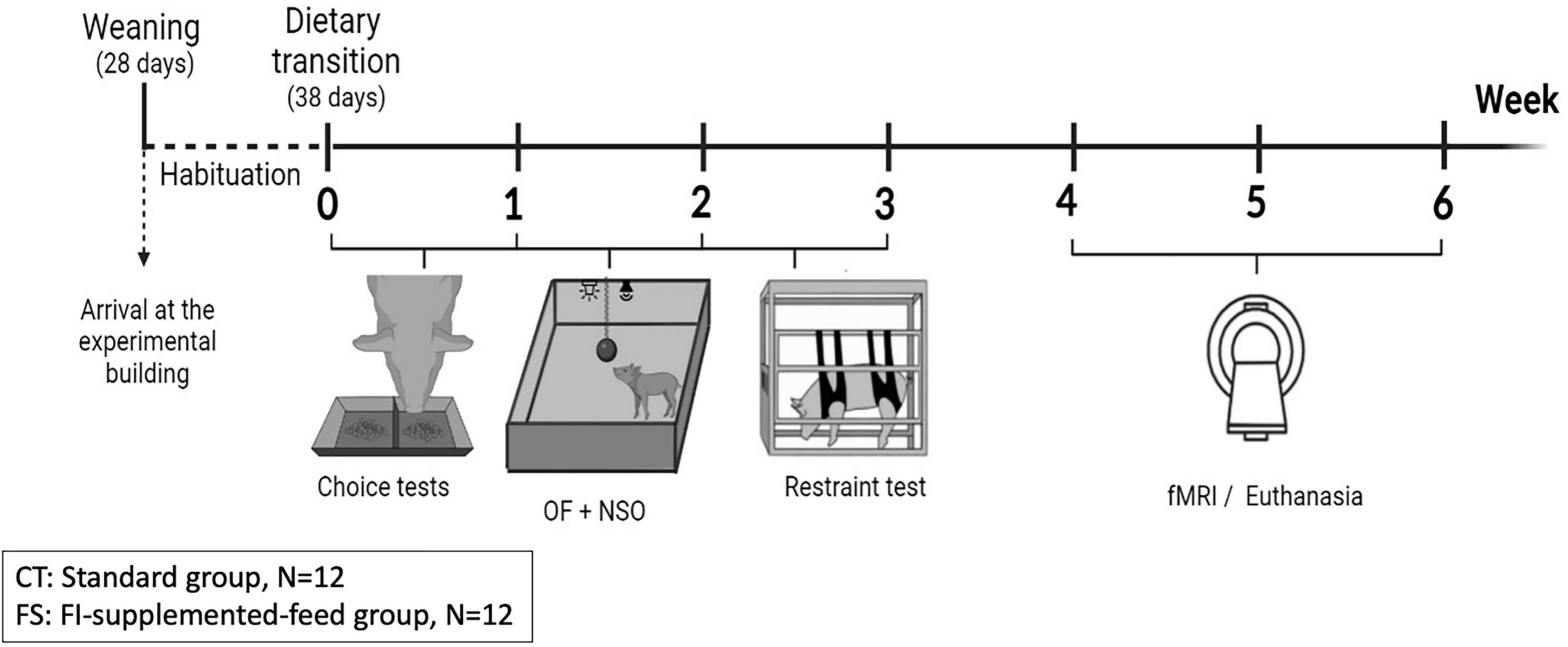
Experimental design to study the effects of a functional food ingredient on feed consumption, preferences, as well as the behavioral and brain responses to acute stress. From their arrival in the experimental building, piglets had 10 days to get habituated to their new environment and to the first-age feed before a dietary transition toward second-age feed composed of different composition, granulometry and texture. The days after this dietary transition, two-choice food preference tests (choice tests) were conducted. Emotional reactivity to acute stress was evaluated through different behavioral tests: Open-field (OF), Novel suddenly moving object (NSO) and physical contention test (restraint test). Brain responses to the functional ingredient and the two different feeds’ odor, as well as to an acute pharmacological stressor (intravenous injection of Synacthen®) were investigated during Week 4 and 5 in habituated and naive animals. Pigs were euthanized after the functional magnetic resonance imaging (fMRI) session.

The entire experimental design is presented in Figure 1.

### Behavioral explorations

#### Body weight and food consumption evolutions

Animals were divided into 2 experimental groups of comparable mean body weight (CT: 7.90 ± 0.52 kg; FS: 7.91 ± 0.55 kg). Pigs had 24-h access to feed except the day before behavioral tests (choice test, OF+ NSO, restraint test, fMRI) where subjects put fasting at 17 h. The daily feed refusals were weighed every day for each, and piglets were weighed weekly from weaning until the end of experimentation. The food consumption was calculated for each experimental group.

#### Two-choice food preference tests

On the day of the feed transition, the animals were subjected to two-choice food preferences tests. These tests were repeated each morning on fasted pigs during 5 consecutive days. Each individual pen was equipped with a bipartite trough in which were dropped two feed rations (600 g the first day then 500 g the next days) of second age feed, one of control feed and the other supplemented with the functional ingredient (FI). The animals were free to eat the control feed (*CF*) or FI-supplemented feed (SF) during the 20 min of testing. During this test, an observer manually noted the first feed tasted, the time spent with the snout over each trough (eating or not) and the number of alternations between troughs. The refusals were weighed at the end of each test to calculate the consumption of each feed. Left and right positions of the feeds in the troughs were counterbalanced across animals and test days to prevent any laterality bias.

#### Open-field test in a novel environment

Open-field (OF) tests were conducted to study emotional reactivity of piglets during social isolation in a new environment (10, 11, 13). Fifteen minutes before the start of the test, fasted animals received 1/3 of their daily ration. Pigs were individually led to the OF arena and waited 30 s at the entrance before entering. The OF test began when the animal had fully entered the room (2.6 × 2.8 m). A camera positioned on the room’s ceiling enabled recording the body postures (standing, lying, sitting, kneeling), locomotion, vocalizations, exploratory, alert, and escape behaviors during the 5-min test, using The Observer XT 10 (Noldus, Wageningen, Netherlands).

#### Reaction to a novel suddenly moving object

The test was conducted to study the behavioral and emotional responses to an acute unpredictable stressor (23, 24) just after the OF test. An unknown object (orange-colored ball) was dropped and jiggled in the center of the OF room concurrently to stressful sounds and lights during 5 s. The ball then stayed suspended just above the floor in the center of the room so that the animal could approach and touch it. The latency of contact with the object and the behaviors observed during the OF test were recorded for 5 min.

#### Physical contention test

This test was adapted from the forced-swimming and tail suspension tests used in rodents to study behavioral despair as a depression-like symptom in pigs (10, 25–27). Fifteen minutes before the start of the test, fasted animals received 1/3 of their daily ration. Pigs were equipped with two suspension harnesses and elevated with an electric-hydraulic system until their feet rose off the ground. The total duration of physical mobility, the number of attempts to escape, and the number of vocalizations were recorded for 5 min. A perseverance index was determined as the average duration of one escape attempt as described in Menneson et al. (11).

### Functional imaging

#### Anesthesia

Pre-anesthesia was performed with an intramuscular injection of Tiletamine/Zolazepam (15 mg/kg — Zoletil 50® 25 mg/ml, Virbac, Carros, France) in overnight-fasted animals. Isoflurane inhalation (Isoflu-Vet 1,000 mg/g® 250 ml, Dechra, Montigny-le-Bretonneux, France) was used to suppress the pharyngotracheal reflex and then establish a surgical level of anesthesia, at 5 and 2–3%, respectively. After intubation, anesthesia was maintained with 2–2.5% Isoflurane and mechanical ventilation allowed adjustment of respiratory frequency at 16 breaths/min with a tidal volume of 380 ml (Fabius, Draeger®, Germany). The tidal volume was adapted in order to target an end-tidal CO2 (EtCO2) between 4 and 5.5%. Fraction of inspired and exhaled Isoflurane was assessed. Heart rate was comprised between 100 and 150 beats per minute. The temperature was recorded with intra-rectal thermal probe. Animals were covered with a blanket during imaging, the temperature was not recorded. The right ear was equipped with a venous route. Cotton wool with an additional headset were used to conceal the animal’s ears, and tape was used to maintain the eyes closed. Animals were euthanised at the end of the imaging session *via* an intravenous injection of T61 (1 ml/10 kg) without awakening from anesthesia.

#### Olfactory stimulation

We used a custom-made olfactory stimulation apparatus already used in previous studies (1, 28), which was located outside the magnet-shielded room. Briefly, animals were equipped with a tube inserted into the right nostril, allowing air circulation in the entire nasal cavity. Three kinds of odorant stimulation have been tested: One based on the control feed odor (*CF*), one corresponding to the functional food ingredient alone (D16729, FI), and one based on the control feed odor combined with the functional food ingredient D16729 (*CF* + FI). The galenic form of D16729 used for brain imaging was especially formulated to be miscible in water in order to control the exact concentration of the ingredient. The control stimulation consisted in the diffusion of non-odorized air.

#### Stimulation paradigm and pharmacological-induced stress

For each animal, a sequence of stimulation consisted in the alternation between odorant stimulation (30 s) and control stimulation (30 s). The control stimulation corresponded to the diffusion of non-odorized air (NO). Three sequences of stimulation were performed before intravenous injection with 2.5 mg/kg of Synacthen®, an ACTH-related agonist that pharmacologically mimics the effects of an acute stress through the activation of the HPA axis (1): One with the control formulation (*CF*), one with the mix of control and functional food ingredient formulation (*CF* + FI) and one with the functional food ingredient alone (Pre-Syn FI). Thirty minutes after the Synacthen® injection, a new stimulation was performed with the functional food ingredient alone (Post-Syn FI).

#### MRI image acquisition

Image acquisition was performed as previously described (28, 29) on a 1.5-T magnet (Siemens Avanto) at the Research Platform for Multimodal Imaging and Spectroscopy (PRISM, Rennes, France). Acquisitions were performed using a combination of coils (Body and Spine surface matrix coils, commercial products from Siemens, 6 channels were used for each) for optimized signal-to-noise ratio acquisition. Gradient shimming was performed automatically. *T1 weighted anatomical image acquisition:* A MP-RAGE sequence was adapted to the adult minipig anatomy (160 slices, 1.2 × 1.2 × 1.2 mm3, NA = 2, TR = 2,400 ms, TE = 3.62 ms, TI = 854 ms, FA = 8°, acquisition duration 15 min). *BOLD (Blood-Oxygen-Level Dependent) signal acquisition:* An echo planar imaging sequence was adapted to pig head geometry (32 slices, TR/TE: 2500/40 ms, FA: 90°, voxel size: 3 mm × 3 mm × 3 mm^3^). The field of view was of 192 × 192 mm, the matrix size was 64 × 64, and the total EPI imaging time was 20 min 25 (490 volumes × 2.5 s/volume, 4 initial volumes as dummy scans). The first two acquired volumes were excluded from the data analysis, meaning that no stimulation was performed during this period. Imaging was performed on 24 animals. Three animals were excluded as we detected a loss of MR signal in the frontal lobe due to the anatomical presence of an air cavity anterior to the brain and 1 animal was excluded because of movements during the anatomical image acquisition inducing poor realignment and segmentation.

### Data analysis and statistical image analysis

#### Statistical behavior analysis

Data were analyzed with the R 3.5.1 Software. Comparisons between groups were made with a two-way ANOVA (group × sex × group*sex). The normality of residuals was tested with Shapiro–Wilk test. In absence of normality, data were log-transformed and analyzed again. Non-parametric tests (Mann–Whitney) were used as a last resort for comparison between groups and sexes. Data are expressed as mean ± SD. Differences were considered significant at *p* < 0.05 and as a trend when 0.05 < *p* < 0.1.

#### Statistical brain image analysis

Data analysis was adapted from Menneson et al. (1) and performed with SPM12 (version 6,914, Wellcome Dept. of Cognitive Neurology, London, United Kingdom) and using Nipype for pipelining (30). Due to limitations related to the size of the pig’s brain and the effect of anesthesia on brain activity, we used a non-standard statistical analysis with regards to human statistical standards usually considering statistical significance at a cluster level with value of *p* < 0.05.

*Voxel-based statistic*: First-level (within-individual contrast) and second-level (within-group contrast) *t*-test statistics were assessed for each voxels comparison with a threshold set at *p* < 0.05 in order to produce the brain activation maps.

*Small Volume Correction (SVC)-based statistic*: Anatomical ROI from the Saikali pig brain atlas [31] were used for SVC-based statistics with a value of *p* corrected for multiple ROIs comparisons with a Bonferroni correction and at a threshold of 0.05 (peak level). The ROI selected were those that were previously found modulated in the context of chronic stress in our pig model (1, 10) and corresponded to bilateral: hippocampus (Hi), amygdala (A), anterior prefrontal cortex (aPFC), dorsolateral prefrontal cortex (dlPFC), ventral anterior cingulate cortex (vaCC) and dorsal anterior cingulate cortex (daCC). We also added to this list the nucleus accumbens (ACC), putamen (PU) and caudate (CAU).

## Results

### Behavioral analyses

All the behavioral data are summarized in Table 1.

**Table 1:** Summary of behavioral outcomes for individuals in the control group (CT) and functional ingredient-supplemented group (FS).

	CT group			FS group			*p*-value
	Control feed	Food intake	*P*-value	Conrol feed	Food intake	*P*-value	
**Choice tests**							
First choice (%)	51.7 ± 24.8	48.3 ± 24.8	1.00	51.7 ± 23.3	48.3 ± 23.23	1.00	
Food intake (g)	67.3 ± 55.6	71.2 ± 58.2	0.42	54.5 ± 57.9	63.3 ± 52.0	0.61	
Time spent above troughs (s)	518.5 ± 130.2	503.8 ± 138.9	0.80	478.0 ± 79.3	591.0 ± 99.1	0.06	
Number of alternations	14.4 ± 9.3			16.4 ± 10.3			0.46
**Open-field**							
Vocalizations	57.5 ± 39.8			53.3 ± 44.8			0.81
Locomotion (s)	154.0 ± 44.5			162.0 ± 21.9			0.59
Periphery area (s)	296.4 ± 15.9			304.4 ± 11.9			0.30
Middle area (s)	8.7 ± 13.5			5.4 ± 5.2			1
Exploration (s)	195.2 ± 74.5			207.5 ± 45.2			0.42
Alert (s)	55.5 ± 63.4			38.8 ± 31.7			0.42
**Novel suddenly moving object**							
Locomotor activity (s)	112.0 ± 34.6			100.4 ± 31.9			0.41
Vocalizations	80.2 ± 50.1			78.75 ± 78.8			0.96
Alert (s)	119.0 ± 54.1			134.7 ± 50.4			0.49
Peripheral area (s)	**289.7 ± 11.4**			**307.6 ± 11.6**			**0.001**
Middle area (s)	**17.2 ± 15.5**			**4.2 ± 5.9**			**0.016**
Environment exploration (s)	105.1 ± 58.2			100.0 ± 49.1			0.83
Object exploration (s)	**5.4 ± 6.1**			**1.1 ± 1.8**			**0.037**
Contact latency (s)	**195.1 ± 85.1**			**281.7 ± 34.4**			**0.004**
Duration of exploration (s)	105.1 ± 58.2			100 ± 49.1			0.83
**Restraint test**							
Duration of mobility (s)	21.2 ± 9.9			24.2 ± 12.5			0.53
First escape latency (s)	60.8 ± 82.9			97.3 ± 80.9			0.28
Attempts to escape	5.4 ± 2.7			7.3 ± 4.3			0.22
Vocalizations	63.6 ± 41.5			69.8 ± 46.8			0.74
Perseverance index	3.7 ± 1.5			3.4 ± 1.6			0.66
Body weight (kg)	27.51 ± 3.36			26.55 ± 2.15			0.88
Average food intake during experimental period (g)	774.1 ± 457.8			791.1 ± 516.1			0.60

Significant differences appear in bold, with a threshold at *p* < 0.05.

#### Body weight and food consumption evolutions

Body weight did not differ between groups at the beginning of the study (CT: 7.90 ± 0.5 kg, FS: 7.91 ± 0.5 kg, *p* = 0.96), and the supplementation did not induce growth difference (weight at the end of the study: CT: 27.51 ± 3.36 kg, FS: 26.55 ± 2.15 kg, *p* = 0.88). Food intake did not differ during the entire experimental period (Table 1; *p* = 0.60).

#### Two-choice food preference tests

Animals did not show food preference in terms of first choice (Table 1; CT group: *CF*: 51.7 ± 24.8%, SF: 48.3 ± 24.8%, *p* = 1.00; FS group: *CF*: 51.7 ± 23.3%, SF: 48.3 ± 23.23%, *p* = 1.00), food intake (CT group: *CF*: 67.3 ± 55.6 g, SF: 71.2 ± 58.2 g, *p* = 0.42; FS group: *CF*: 54.5 ± 57.9 g, SF: 63.3 ± 52.0 g, *p* = 0.61), number of alternations between the troughs (CT: 14.4 ± 9.3 switches, FS: 16.4 ± 10.3 switches, *p* = 0.46) and the time spent above these troughs in CT (*CF*: 518.5 ± 130.2 s, SF: 503.8 ± 138.9 s, *p* = 0.80) and FS animals. Only animals from the FS group had a trend to spending more time above the FI-supplemented feed (*CF*: 478.0 ± 79.3 s, SF: 591.0 ± 99.1 s, *p* = 0.06).

#### Openfield test in a novel environment

Behavioral parameters did not differ between CT and FS in the non-familiar environment (Table 1): number of vocalizations (CT: 57.5 ± 39.8, FS: 53.3 ± 44.8, *p* = 0.81), duration of movement (CT: 154.0 ± 44.5 s, FS: 162.0 ± 21.9 s, *p* = 0.59), time spent at the periphery (CT: 296.4 ± 15.9 s, FS: 304.4 ± 11.9 s, *p* = 0.30) and middle area (CT: 8.7 ± 13.5 s, FS: 5.4 ± 5.2 s, *p* = 1), duration of exploration (CT: 195.2 ± 74.5 s, FS: 207.5 ± 45.2 s, *p* = 0.42) and alert (CT: 55.5 ± 63.4 s, FS: 38.8 ± 31.7 s, *p* = 0.42).

#### Reaction to a novel suddenly moving object

**Figure 2:**
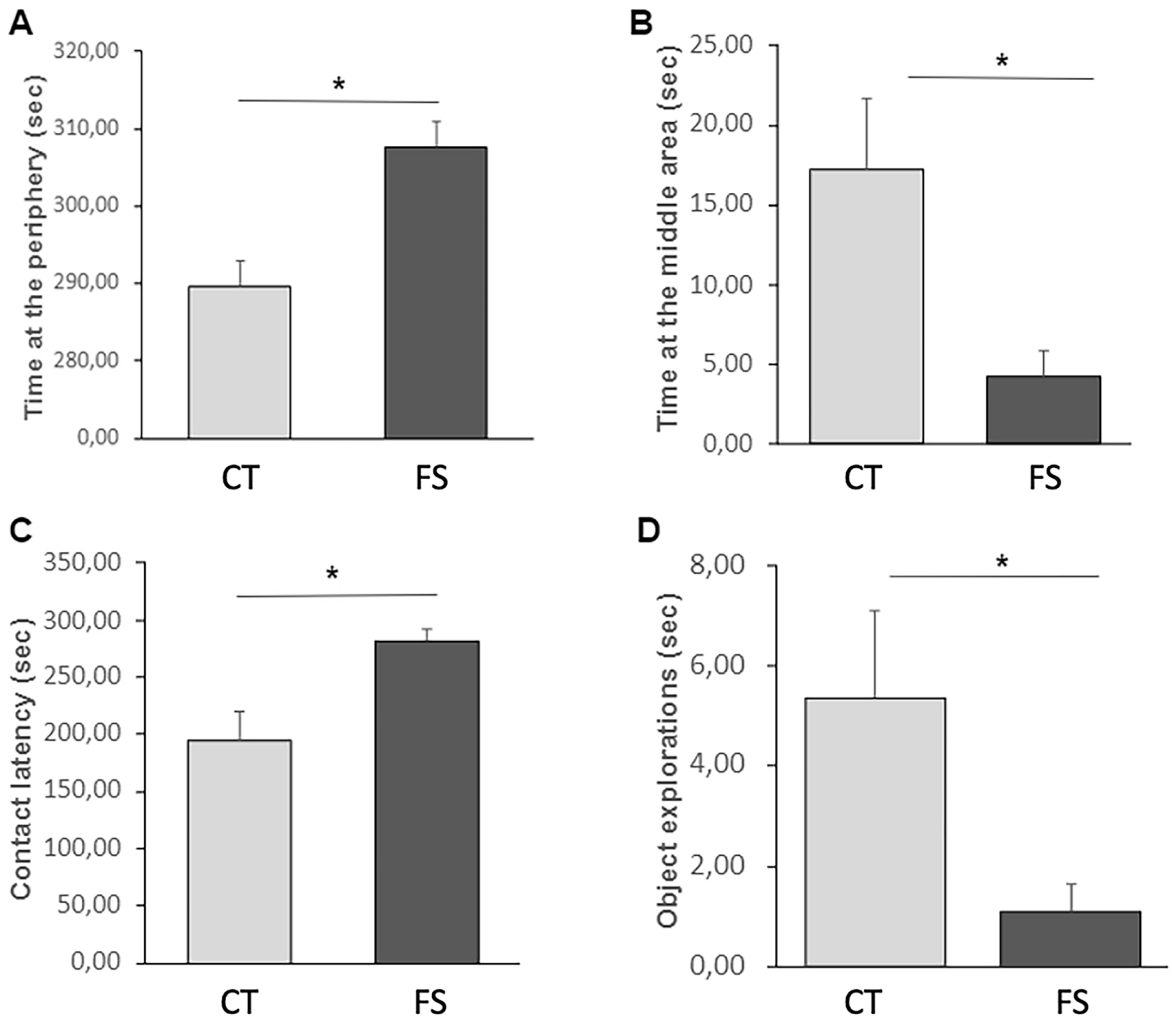
Behavioral comparisons in the NSO between animals fed with a control feed (CT, *n* = 12) and animals fed with a feed supplemented with the functional ingredient (FS, *n* = 12). FS animals spent more time than CT animals at the periphery of the test arena **(A)** and less time in the middle area **(B)** where the object was suspended. They also presented a higher contact latency **(C)** with the object and explored it less than CT **(D)**. Mean ± SD, two-way ANOVA, *p < 0.05.

There was no difference detected between groups in level of locomotor activity (Table 1; CT: 112.0 ± 34.6 s, FS: 100.4 ± 31.9 s, *p* = 0.41), number of vocalizations (CT: 80.2 ± 50.1, FS: 78.75 ± 78.8, *p* = 0.96), duration of environment exploration (CT: 105.1 ± 58.2 s, FS: 100.0 ± 49.1 s, *p* = 0.83) and time on alert (CT: 119.0 ± 54.1 s, FS: 134.7 ± 50.4 s, *p* = 0.49). But the time at the periphery and middle area of the test arena, contact latency and duration with object exploration differ between CT and FS animals (Figure 2).

#### Physical contention test

No differences could be detected in the physical contention test (Table 1). The duration of mobility (CT: 21.2 ± 9.9 s, FS: 24.2 ± 12.5 s, *p* = 0.53), first escape latency (CT: 60.8 ± 82.9 s, FS: 97.3 ± 80.9 s, *p* = 0.28), number of attempts to escape (CT: 5.4 ± 2.7, FS: 7.3 ± 4.3, *p* = 0.22), number of vocalizations (CT: 63.6 ± 41.5, FS: 69.8 ± 46.8, *p* = 0.74) and perseverance index (CT: 3.7 ± 1.5, FS: 3.4 ± 1.6, *p* = 0.66) did not differ significantly between groups.

### Brain imaging analyses

Data from the SVC analyses are provided as Supplementary Table S1. The following overall contrast-by-contrast descriptions correspond to the whole-brain analyses.

#### Brain responses to the feed odor in relation to FI perception and familiarity

**Figure 3:**
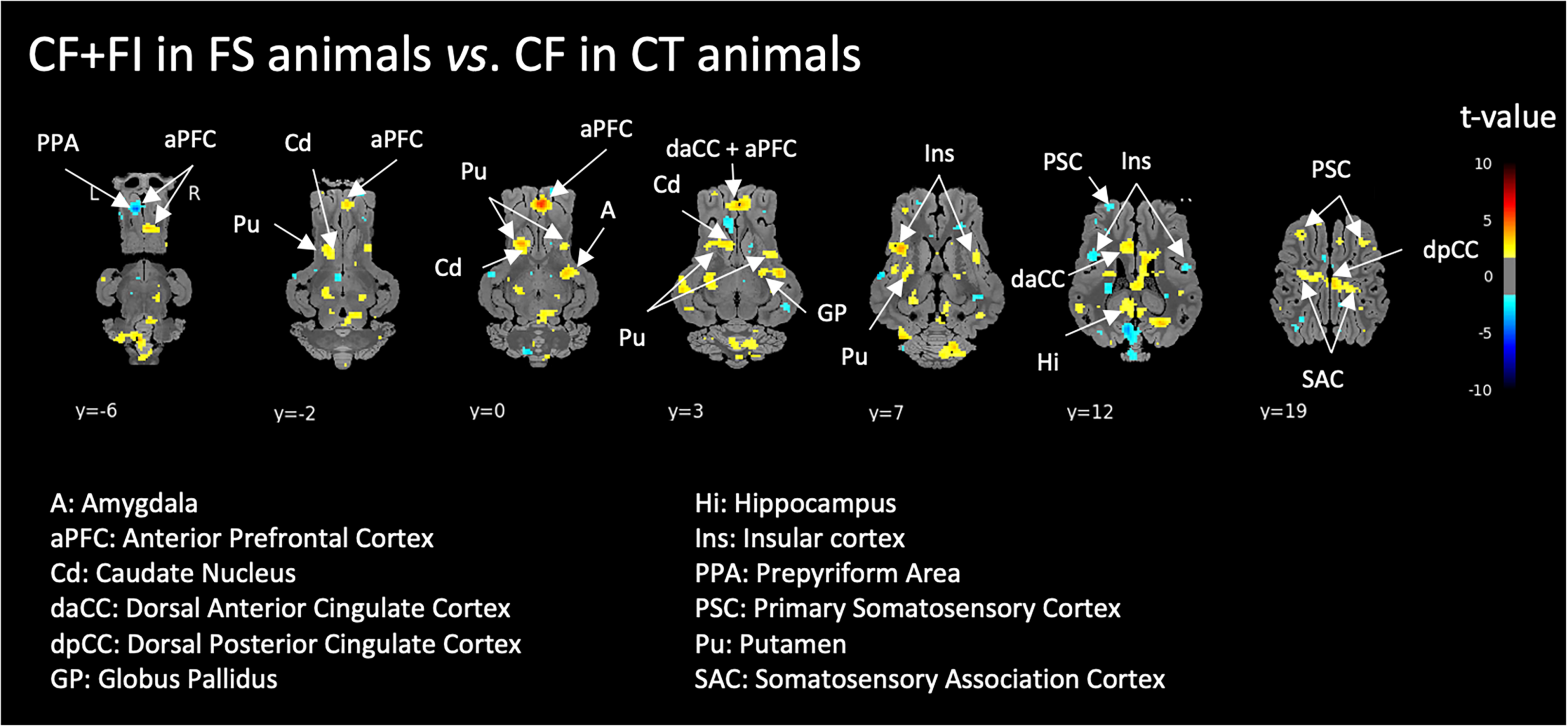
Brain responses to the control feed odor (*CF*) supplemented with the functional ingredient (FI) in FI-habituated animals (FS) compared to the brain responses to the control feed odor (*CF*) in control animals (CT). Horizontal maps of brain BOLD responses are represented at different dorsal-ventral levels (*y*) related to the posterior commissure (in mm). Value of *p* threshold = 0.05, *k* > 20 (number of voxels per cluster).

When comparing the perception of two familiar feed odors, the whole-brain analyses revealed significantly increased activations in the corticostriatal circuit (prefrontal and cingulate cortex, caudate, putamen and globus pallidus) as well as in the hippocampus and amygdala of FS animals exposed to the *CF* + FI compared to CT animals exposed to *CF* (Figure 3).

#### Brain responses to FI in naive CT animals vs. FS animals

**Figure 4:**
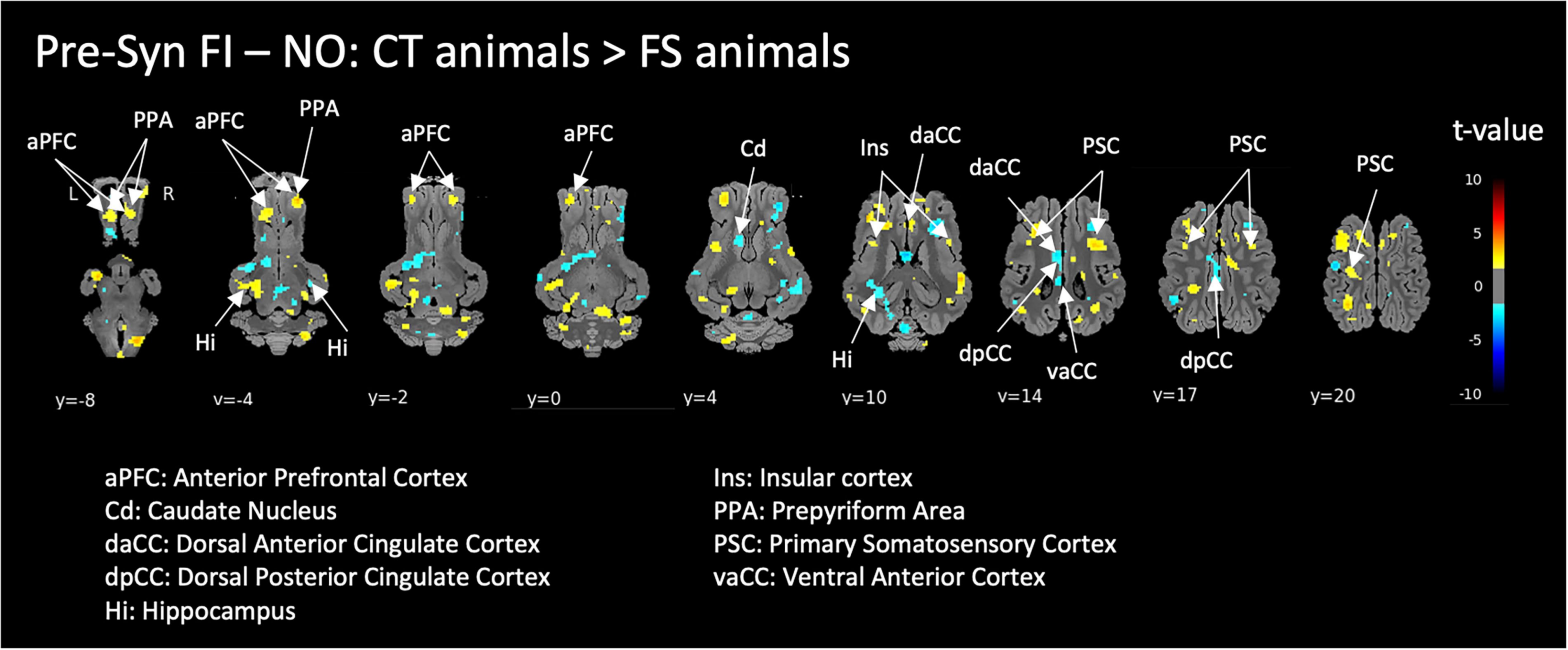
Responses to the functional ingredient (FI compared to non-odorized air, NO) in naive control animals (CT) compared to FI-habituated animals (FS) before (Pre-Syn) induction of the acute pharmacological stress (injection of Synacthen®). Horizontal maps of brain BOLD responses are represented at different dorsal-ventral levels (*y*) related to the posterior commissure (in mm). Value of *p* threshold = 0.05, *k* > 20 (number of voxels per cluster).

The perception of the functional ingredient (FI) in naive control animals (CT) compared to FI-habituated animals (FS) induced activation in the anterior prefrontal cortex, prepyriform area and bilateral insula, deactivation in the caudate, as well as ambivalent responses (either activation or deactivation depending on the area’s subdivisions) in the cingulate cortex and hippocampus (Figure 4).

#### Brain responses to a pharmacological acute stress

**Figure 5:**
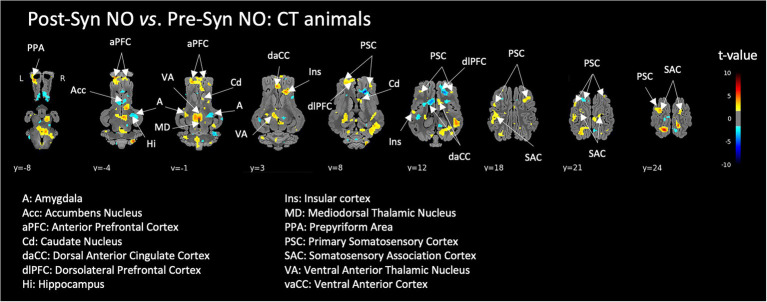
Brain responses of control animals (CT) to the induction of a pharmacological acute stress (injection of Synacthen®), corresponding to the contrast ‘after – before’ injection (i.e., Post-Syn – Pre-Syn). BOLD responses during the stimulation with non-odorized air (NO) were used for this comparison. Horizontal maps of brain BOLD responses are represented at different dorsal-ventral levels (y) related to the posterior commissure (in mm). Value of *p* threshold = 0.05, *k* > 20 (number of voxels per cluster).

A pharmacological acute stress in control animals (CT) induced the activation of a brain network including the anterior prefrontal cortex, dorsal anterior cingulate, insula, somatosensory cortex areas and thalamic nuclei. Ambivalent brain responses (either activation of deactivation depending on the hemisphere or area’s subdivisions) were observed in the amygdala and striatum (Figure 5).

#### Effect of chronic exposure to FI on the brain responses to acute stress

**Figure 6:**
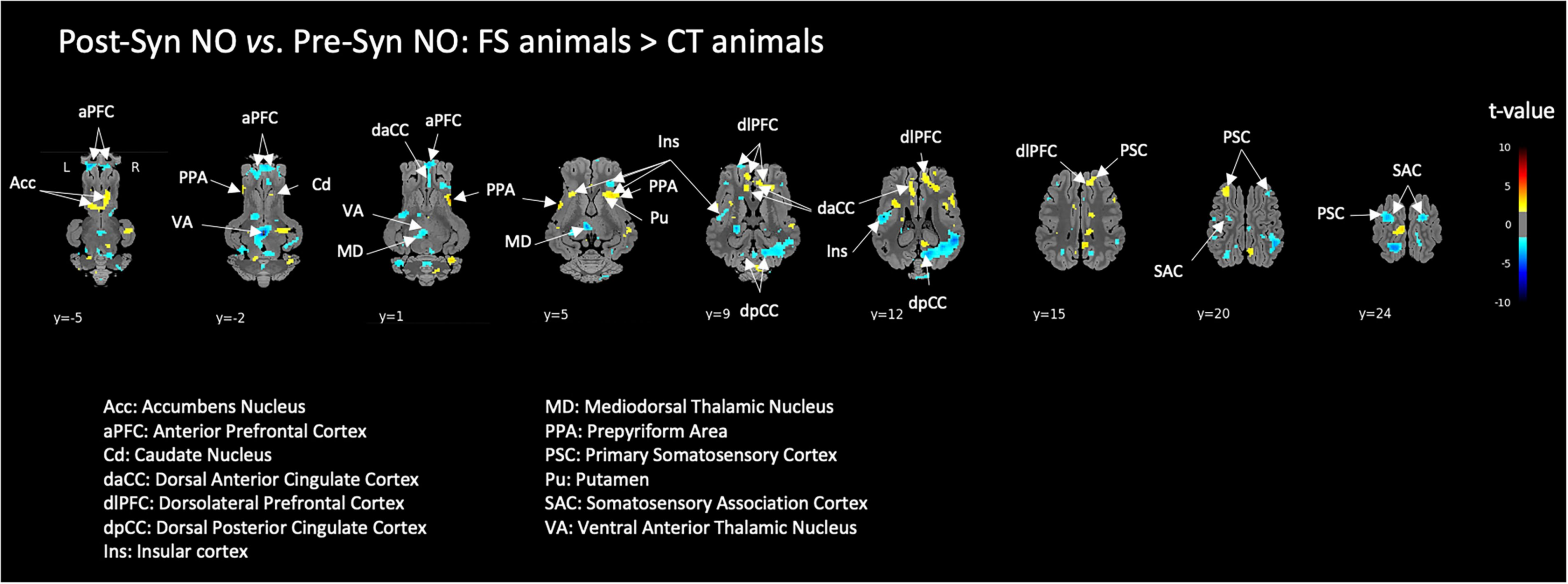
Effect of previous chronic exposure to the functional food ingredient on the brain responses to a pharmacological acute stress. Brain responses of FI-habituated animals (FS) were compared to those of control animals (CT), ‘after – before’ the acute stress (i.e., Post-Syn – Pre-Syn). BOLD responses during the stimulation with non-odorized air (NO) were used for this comparison. Horizontal maps of brain BOLD responses are represented at different dorsal-ventral levels (*y*) related to the posterior commissure (in mm). Value of *p* threshold = 0.05, *k* > 20 (number of voxels per cluster).

Many brain activations induced by the pharmacological acute stress induced during the fMRI session were significantly lower in animals that were previously exposed to the functional ingredient (FS) compared to naive control animals (CT). This was notably the case for several prefrontal subdivisions, the insula and thalamic nuclei. Interestingly, higher activation of the striatum was observed in FS animals compared to CT animals (Figure 6). Though, there was no difference between groups in the hippocampus and amygdala.

#### Effect of acute exposure to FI on the brain responses to acute stress in naive animals (CT)

**Figure 7:**
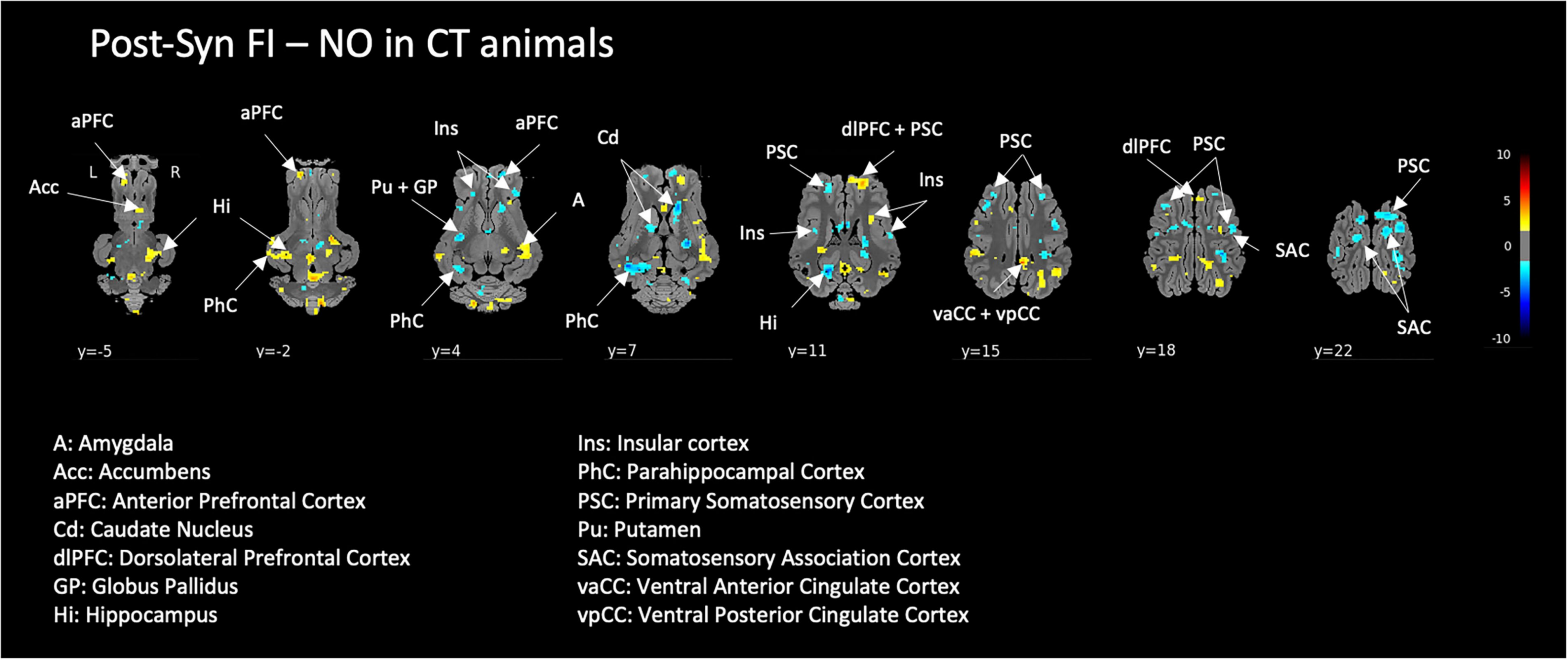
Effect of the exposure to the functional food ingredient (FI compared to non-odorized air, NO) on the brain responses to a pharmacological acute stress in naive control (CT) animals. Horizontal maps of brain BOLD responses are represented at different dorsal-ventral levels (*y*) related to the posterior commissure (in mm). Value of *p* threshold = 0.05, *k* > 20 (number of voxels per cluster).

Exposure to FI in the context of acute pharmacological stress in naive animals modulated a large set of brain structures including prefrontal and cingulate cortical areas, the striatum, insula, and somatosensory areas. Interestingly, the hippocampus and parahippocampal cortex showed ambivalent responses, i.e., either activated or deactivated according to the subdivisions (Figure 7).

## Discussion

### Main results

This study investigated the ability of a new functional food ingredient (FI) to reduce behavioral and brain responses to acute stress, induced either during dedicated behavioral tests or pharmacologically with an intravenous injection of Synacthen®, in individuals that were not subjected to chronic psychosocial stress contrary to what was previously investigated (1, 10, 11). Overall, our results showed little differences between groups during the behavioral tests. FS animals chronically exposed to the FI tended to spend more time above the functional feed and spent significantly more time at the periphery of the openfield arena. The latency to contact the novel object was longer and FS animals spent less time exploring the object compared to CT animals. Neuroimaging data demonstrated that the frontostriatal and limbic responses to the FI were influenced by previous exposure to FI, and that the activation of these circuits was higher in FS animals exposed to the functional feed odor compared to CT animals exposed to a similarly familiar feed odor without FI. The pharmacological acute stress induced during imaging provoked significant brain activations in the prefrontal and thalamic areas as well as ambivalent responses in the amygdala and striatum. Compared to CT animals, the stress-induced prefrontal and thalamic activations were alleviated in FS animals, which also showed more activity in the nucleus accumbens and dorsal striatum. Finally, the acute exposure to FI in naive animals modulated their brain responses to acute pharmacological stress. Overall, these results showed how previous habituation to this new FI can modulate the brain areas involved in food pleasure and motivation while alleviating the brain responses to acute stress.

### Behavioral outcomes of chronic exposure to the functional ingredient

On the one hand, chronic exposure to the FI did not induce a food preference for FI-supplemented feed (SF). This result can be explained by the fact that, within each group, the piglets had individual preferences but not for the same feed. Our results, like those of Clouard et al. (32), illustrate the impact of individual variability on food preferences. Many factors impact this variability such as maternal and social influences (33, 34). An increase in the number of individuals could have helped to compensate for this variability. It is difficult to compare the effects of FI between studies due to discrepancies in the methodologies used (simple natural substance, complex mixture, dosage). Paës et al. (35) found that in a two-choice food preference test, rabbits had a moderate preference for vanilla flavored gel, suggesting that vanillin can have an effect on food preferences.

On the other hand, exposure to the FI did not alleviate the contention stress and fear reactions toward a novel moving object. FS animals were even more cautious than CT animals toward the novel moving object. In view of the literature, these results are surprising as vanillin have repeatedly shown antidepressant effects in rodent models (14, 16, 17, 36, 37). We can hypothesize that under acute stress our formulation and/or dosing may have not been sufficient to reduce significantly the emotional reactivity of FS animals. Using another FI, we have already described different behavioural outcomes depending on the dose used (38) and further experiments might be dedicated to test this novel FI at different doses. Another hypothesis is that chronic exposure to the FI increased awareness and prudence toward the novel moving object. Vanillin has been shown to have nootropic effects in mice. For example, Anand et al. (39) demonstrated that vanillin significantly reversed the memory and behavioral deficits caused by scopolamine, since significant improvement in memory were observed in negative reinforcement and elevated plus maze, which are stressing conditions for mice. It is still not clear whether vanillin might also induce increased vigilance or fear during a stressing event, as observed in our pig model, but perhaps attention toward stressors is modulated as part of the cognitive processes necessary for learning and memorization. Further behavioral tests might be performed in our model to specifically investigate attention and memory processes, notably in stressing conditions.

### Brain responses to the feed odor in relation to FI perception and familiarity

Exposure to the odor of the feed supplemented with the FI induced specific brain activations in the frontostriatal and limbic systems of FS animals independently from feed familiarity, which is in line with a previous study (40). Indeed, when two similarly familiar feeds are compared, i.e., FI feed odor in FS animals vs. control feed odor in CT animals, differences of brain activation in the corticostriatal and limbic circuits indicate that the neuromodulating effect of FI is not only related to the fact that FS animals were habituated to it. Such results therefore indicate an “added value” of the FI present in the feed for brain modulation.

### Brain responses to FI in CT animals vs. FS animals

Acute exposure to the FI in naive CT compared to FS animals modulated brain activity in the anterior prefrontal cortex, olfactory cortex, cingulate cortex and hippocampus, a brain network involved in sensory, cognitive and emotional valuation/memorization. Higher activation of brain areas treating food sensory stimuli (prepyriform area, insula) is quite logical since the FI odor represents a new olfactory stimulus for CT animals that were always housed separately from the FS animals not to “pollute” their environment atmosphere. The anterior prefrontal cortex is involved in anticipating events in the environment or managing emotional reactions, which are important functions for the treatment of novel odors or food stimuli (41). Deactivation of the caudate might be related to neophobia towards this novel odor, a well-known phenomenon (32), since the caudate plays a role both in appetitive motivation and aversive responses to novelty (42, 43). Finally, the modulation of the hippocampus and cingulate cortex might be related to their role in learning, memory and specific attention. We have previously demonstrated in pigs for example that the hippocampus is activated by new food odors and that these responses are decreased in the context of a psychosocial stress (10). Even though none of our animals were subjected to this treatment, the naivety of our CT animals towards the FI might explain their hippocampal responses.

### Brain responses to a pharmacological acute stress

The pharmacological stress induced during imaging provoked significant brain activations in the prefrontal, insular, cingulate and thalamic areas as well as ambivalent responses in the amygdala and striatum, which is coherent with a previous study where we first implemented in pigs our pharmacological fMRI procedure with intravenous injection of an ACTH-agonist (1). This ‘firing’ stressed brain is consequently a consistent and repeatable result that might be generalized as a model of acute pharmacological stress for brain imaging or other types of explorations. Some authors documented the amygdala reactivity during cortisol reactivity to a psychosocial stressor (44) while others showed that increased perceived stress is directly associated with increased amygdala connectivity with frontal cortical regions (45). The prefrontal and cingulate cortices also play a major role in acute perceived stress and cortisol responses (46). Our pig model consequently represents a real asset to investigate the effects of potential interventional strategies on the brain responses to acute stress.

### Effects of chronic or acute exposure to FI on the brain responses to acute stress

Compared to CT animals, most of the stress-induced prefrontal and thalamic activations were alleviated in FS animals. Comparable results have already been obtained with another functional food ingredient, with a major decrease of the stress-induced brain activity in animals chronically exposed to the FI (1). A novel result in the present study is the higher activity of the nucleus accumbens (i.e., hedonic hotspot) and dorsal striatum (putamen and caudate) in FI-habituated animals (FS). The former ingredient used by Menneson et al. (1) contained mainly *Citrus sinensis* extracts and plant emulsifying agents. The FI used in the present study contained vanillin as well as furaneol, diacetyl and a blend of aromatic fatty acids, whose effects are still documented on stress adaptation and food pleasure/anticipation (two main functions targeted with this FI). A few hypotheses about the action mechanisms of these molecules have been presented in the introduction and will not be discussed here again since our functional ingredient was a complex blend, which prevents debating the respective effects of its different components in the context of our own results.

When looking at the brain responses of CT animals when they were exposed to the FI during the pharmacological acute stress, the results are not comparable to those obtained in FS animals that were chronically exposed to the FI. Notably, the acute perception of FI in naive animals did not reduce the strong activations induced in the prefrontal and thalamic regions, suggesting that habituation to the FI is necessary to alleviate the brain responses to stress and that only acute exposure to FI is not sufficient. Interestingly, a particularity appeared in CT animals when exposed to the FI during stress, i.e., strong modulations of the hippocampal structures, which corroborates what has been shown before the pharmacological stress induction in these animals. The perception and sensory treatment of the FI, which is a novel odor for CT animals, probably elicits learning and memory processes supported by hippocampal structures.

### Limitations of the study

As stated before, our functional ingredient is a complex blend composed of several active molecules, which can have additive or synergetic effects. It is consequently difficult to disentangle the respective weight of these molecules in the observed behavioral and neurological outcomes. The functional food ingredient showed little effect on behavioral responses. It is probable that the tests used to induce an acute stress were not stressful enough, provoking a short-lasting surprise rather than a real stress in the context of the NSO test for example. This highlights the necessity to identify or design new behavioral tests that would better illustrate or correlate with our brain imaging data. The use of this novel food ingredient in pig breeding facilities at a large scale would also provide interesting field data on the animals’ general behavior, health, welfare and *in fine* production. Indeed, since there is a high interindividual variability in the outcomes of behavioral tests, small groups of only 12 animals limit the possibility to observe clear-cut differences between treatments. Finally, the interesting results observed at the brain level were obtained in animals chronically fed with a single feed supplemented with the functional ingredient. A possible transposition of these results to humans would necessitate a deep consideration about the administration mode and dose.

## Conclusion

Despite few differences at the behavioral level, we were able to observe that, independently of the familiarity of the food, the active substance of the FI could modulate the brain activity in the frontostriatal and limbic systems of the animals through the olfactory pathway. Our acute pharmacological stress model allowed us to observe significant brain activations in regions related to emotional processing, sensory perception, arousal and associative learning. In animals previously exposed to the FI, this stress-induced brain firing was attenuated, which highlights the potential role of this FI on stress adaptation. Further studies are needed to identify the respective effects of each active molecule contained in our food ingredient, and to investigate their effects in the context of better behavioral tests of acute stress in the pig model.

## Data availability statement

The original contributions presented in the study are publicly available. This data can be found at: https://doi.org/10.57745/WE8AX8.

## Ethics statement

The animal study was reviewed and approved by The Regional Ethics Committee in animal Experiment of Brittany and the French Ministry of Higher Education and Research have approved and authorized the entire procedure described in this manuscript (project number APAFIS #31372-2021043015211971_v2).

## Author contributions

DV-L, NC, and YS: experimental design. YS, EB, PD, and NC: technical development. VN and PE: functional ingredient conception and provision. EB, PD, YS, RJ, and P-AE: performing the experiments. EB, YS, and DV-L: data analysis. EB and DV-L: manuscript writing. All co-authors: manuscript revising. All authors contributed to the article and approved the submitted version.

## Funding

This study was funded by Phodé (Terssac, France, https://www.phode.com/en/) and INRAE (https://www.inrae.fr). Phodé provided the functional ingredient tested and support in the form of salaries for VN and PE but did not have any additional role in the study design, data, collection and analysis, decision to publish, or preparation of the manuscript. The specific roles of the authors affiliated to our private partner are articulated in the ‘Author contributions’ section.

## Conflict of interest

Authors VN and PE were employed by Phodé, who provided the functional ingredient and also co-funded the study.

The remaining authors declare that the research was conducted in the absence of any commercial or financial relationships that could be construed as a potential conflict of interest.

## Publisher’s note

All claims expressed in this article are solely those of the authors and do not necessarily represent those of their affiliated organizations, or those of the publisher, the editors and the reviewers. Any product that may be evaluated in this article, or claim that may be made by its manufacturer, is not guaranteed or endorsed by the publisher.
