# Supplementary material for: Exposure to a sensory functional ingredient in the pig model modulates the blood-oxygen-level dependent brain responses to food odor and acute stress during pharmacological MRI in the frontostriatal and limbic circuits
Source: Front Nutr. 2023 Feb 28;10:1123162. doi: 10.3389/fnut.2023.1123162 (PMC10012862; doi:10.3389/fnut.2023.1123162)
Supplement: Supplementary file 1 [file Table_1.DOCX]

**Supplementary Table 1.** Small Volume Correction (SVC) analyses performed on a set of regions of interest (ROI) for each of the contrasts considered. R, right; L, left; Number of voxels in the cluster, k>20; t-value, T; The x,y,z coordinates are indicated in the CA-CP (*commissura anterior-commissura posterior*) plane. A p-value of p<0.0027 was chosen as the threshold for significance after Bonferroni correction (for 18 selected ROIs).

| **CF+FI in FS animals *vs*. CF in CT animals (Figure 3)** | | | | | | | | | |
| --- | --- | --- | --- | --- | --- | --- | --- | --- | --- |
|  |  | CF+FI in FS animals > CF in CT animals | | | | CF in CT animals > CF+FI in FS animals | | | |
| ROI | L/R | k | T | p (uncor) | x, y, z | k | T | p (uncor) | x, y, z |
| ACC | R |  |  |  |  |  |  |  |  |
|  | L |  |  |  |  |  |  |  |  |
| CAU | R | 63 | 3.50 | 0.0013 | 5 22 2 |  |  |  |  |
|  | L | 37 | 3.37 | 0.0017 | -8 9 9 |  |  |  |  |
| PU | R | 95 | 3.93 | 0.00049 | 7 21 0 |  |  |  |  |
|  | L |  |  |  |  |  |  |  |  |
| Hi | R |  |  |  |  |  |  |  |  |
|  | L |  |  |  |  |  |  |  |  |
| A | R |  |  |  |  |  |  |  |  |
|  | L | 28 | 3.19 | 0.00253 | -16 9 2 |  |  |  |  |
| aPFC | R | 71 | 5.11 | 0.00003 | -2 38 0 | 73 | 5.15 | 0.00003 | 3 35 -6 |
|  | L | 34 | 5.09 | 0.00003 | 0 37 0 | 27 | 3.80 | 0.00066 | -7 42 -1 |
| dlPFC | R |  |  |  |  |  |  |  |  |
|  | L |  |  |  |  |  |  |  |  |
| vaCC | R |  |  |  |  |  |  |  |  |
|  | L |  |  |  |  |  |  |  |  |
| daCC | R | 33 | 4.87 | 0.00006 | 0 36 1 |  |  |  |  |
|  | L | 50 | 5.51 | 0.00001 | -1 37 0 |  |  |  |  |
|  |  |  |  |  |  |  |  |  |  |
| **Pre-Syn FI – NO: CT animals > FS animals (Figure 4)** | | | | | | | | | |
|  |  | Pre-Syn FI – NO: CT animals > FS animals | | | | Pre-Syn FI – NO: FS animals > CT animals | | | |
| ROI | L/R | k | T | p (uncor) | x, y, z | k | T | p (uncor) | x, y, z |
| ACC | R |  |  |  |  |  |  |  |  |
|  | L |  |  |  |  |  |  |  |  |
| CAU | R |  |  |  |  |  |  |  |  |
|  | L | 51 | 3.74 | 0.0007 | -5 21 7 |  |  |  |  |
| PU | R |  |  |  |  |  |  |  |  |
|  | L |  |  |  |  | 28 | 3.16 | 0.0027 | -11 17 6 |
| Hi | R |  |  |  |  |  |  |  |  |
|  | L |  |  |  |  |  |  |  |  |
| A | R |  |  |  |  | 44 | 3.11 | 0.0030 | 11 10 -2 |
|  | L |  |  |  |  |  |  |  |  |
| aPFC | R |  |  |  |  |  |  |  |  |
|  | L | 69 | 3.88 | 0.0005 | -8 38 -4 |  |  |  |  |
| dlPFC | R | 80 | 3.66 | 0.0009 | 10 33 7 |  |  |  |  |
|  | L |  |  |  |  | 9 | 3.25 | 0.0022 | -12 35 4 |
| vaCC | R |  |  |  |  | 19 | 3.70 | 0.0008 | 1 14 11 |
|  | L |  |  |  |  |  |  |  |  |
| daCC | R |  |  |  |  | 16 | 3.10 | 0.0031 | 0 14 12 |
|  | L |  |  |  |  |  |  |  |  |
|  |  |  |  |  |  |  |  |  |  |
| **Post-Syn NO *vs*. Pre-Syn NO: CT animals (Figure 5)** | | | | | | | | | |
|  |  | Post-Syn NO > Pre-Syn NO | | | | Pre-Syn NO > Post-Syn NO | | | |
| ROI | L/R | k | T | p (uncor) | x, y, z | k | T | p (uncor) | x, y, z |
| ACC | R |  |  |  |  | 30 | 4.37 | 0.0009 | 2 19 -5 |
|  | L |  |  |  |  | 43 | 4.15 | 0.0012 | -3 19 -5 |
| CAU | R |  |  |  |  | 8 | 4.04 | 0.0015 | 6 17 10 |
|  | L |  |  |  |  |  |  |  |  |
| PU | R |  |  |  |  |  |  |  |  |
|  | L |  |  |  |  |  |  |  |  |
| Hi | R |  |  |  |  |  |  |  |  |
|  | L |  |  |  |  | 32 | 3.54 | 0.0031 | -13 1 -7 |
| A | R |  |  |  |  |  |  |  |  |
|  | L |  |  |  |  |  |  |  |  |
| aPFC | R | 251 | 5.49 | 0.0002 | 6 38 -2 |  |  |  |  |
|  | L | 37 | 4.77 | 0.0005 | -11 30 2 |  |  |  |  |
| dlPFC | R |  |  |  |  |  |  |  |  |
|  | L |  |  |  |  | 91 | 8.29 | 0.000008 | -8 28 9 |
| vaCC | R |  |  |  |  |  |  |  |  |
|  | L |  |  |  |  |  |  |  |  |
| daCC | R | 79 | 5.44 | 0.0002 | 2 34 3 | 34 | 4.48 | 0.00077 | 4 19 12 |
|  | L |  |  |  |  |  |  |  |  |
|  |  |  |  |  |  |  |  |  |  |
| **Post-Syn NO *vs*. Pre-Syn NO: FS animals > CT animals (Figure 6)** | | | | | | | | | |
|  |  | Post-Syn NO *vs*. Pre-Syn NO: | | | | Post-Syn NO *vs*. Pre-Syn NO: | | | |
|  |  | FS animals > CT animals | | | | CT animals > FS animals | | | |
| ROI | L/R | k | T | p (uncor) | x, y, z | k | T | p (uncor) | x, y, z |
| ACC | R |  |  |  |  |  |  |  |  |
|  | L |  |  |  |  |  |  |  |  |
| CAU | R |  |  |  |  |  |  |  |  |
|  | L |  |  |  |  |  |  |  |  |
| PU | R |  |  |  |  |  |  |  |  |
|  | L |  |  |  |  |  |  |  |  |
| Hi | R |  |  |  |  |  |  |  |  |
|  | L |  |  |  |  |  |  |  |  |
| A | R |  |  |  |  |  |  |  |  |
|  | L |  |  |  |  |  |  |  |  |
| aPFC | R | 5 | 3.04 | 0.0035 | 2 25 8 | 127 | 3.46 | 0.0014 | 0 42 0 |
|  | L | 32 | 3.43 | 0.0015 | -3 25 -8 | 136 | 3.72 | 0.0008 | -1 41 0 |
| dlPFC | R |  |  |  |  |  |  |  |  |
|  | L | 108 | 3.20 | 0.0025 | -7 28 11 | 5 | 3.19 | 0.0025 | -3 42 3 |
| vaCC | R |  |  |  |  |  |  |  |  |
|  | L |  |  |  |  |  |  |  |  |
| daCC | R |  |  |  |  |  |  |  |  |
|  | L |  |  |  |  |  |  |  |  |
|  |  |  |  |  |  |  |  |  |  |
| **Post-Syn FI – NO in CT animals (Figure 7)** | | | | | | | | | |
|  |  | Post-Syn FI > NO | | | | Post-Syn FI < NO | | | |
| ROI | L/R | k | T | p (uncor) | x, y, z | k | T | p (uncor) | x, y, z |
| ACC | R |  |  |  |  |  |  |  |  |
|  | L |  |  |  |  |  |  |  |  |
| CAU | R |  |  |  |  |  |  |  |  |
|  | L |  |  |  |  |  |  |  |  |
| PU | R |  |  |  |  | 29 | 3.83 | 0.0020 | 14 10 4 |
|  | L |  |  |  |  |  |  |  |  |
| Hi | R |  |  |  |  |  |  |  |  |
|  | L |  |  |  |  |  |  |  |  |
| A | R |  |  |  |  |  |  |  |  |
|  | L |  |  |  |  |  |  |  |  |
| aPFC | R | 61 | 3.76 | 0.0022 | 6 40 -2 | 6 | 3.49 | 0.0034 | 1 41 -2 |
|  | L |  |  |  |  | 35 | 3.96 | 0.0016 | -7 41 3 |
| dlPFC | R |  |  |  |  |  |  |  |  |
|  | L | 81 | 4.59 | 0.0006 | -7 35 9 | 2 | 3.75 | 0.0022 | -1 15 12 |
| vaCC | R |  |  |  |  | 13 | 3.93 | 0.0017 | 0 15 11 |
|  | L | 3 | 3.78 | 0.0022 | -1 -3 15 | 7 | 4.43 | 0.0008 | -1 15 11 |
| daCC | R |  |  |  |  | 8 | 4.03 | 0.0015 | 0 15 12 |
|  | L |  |  |  |  |  |  |  |  |
